# Lipoprotein insulin resistance score and branched-chain amino acids increase after adrenalectomy for unilateral aldosterone-producing adenoma: a preliminary study

**DOI:** 10.1007/s12020-020-02235-2

**Published:** 2020-03-04

**Authors:** Christian Adolf, Annika M. A. Berends, Margery A. Connelly, Martin Reincke, Robin P. F. Dullaart

**Affiliations:** 1 Medizinische Klinik und Poliklinik IV, Klinikum der Universität München, 80336 Munich, Germany; 2 Department of Endocrinology, University of Groningen, University Medical Center Groningen, Groningen, The Netherlands; 3 Laboratory Corporation of America Holdings (LabCorp), Morrisville, NC USA

**Keywords:** Adrenalectomy, Branched-chain amino acids, Lipoprotein insulin resistance index, Primary aldosteronism, Type 2 diabetes risk

## Abstract

**Background and aims:**

Primary aldosteronism (PA) due to unilateral aldosterone-producing adenoma (APA) is preferentially treated by unilateral adrenalectomy (ADX), but little is known about the changes in lipid and glucose metabolism that may occur after ADX.

**Methods:**

We studied 19 non-diabetic patients who did not use lipid-lowering drugs with PA due to APA before and 6 months after unilateral ADX. Fasting plasma lipids, lipoprotein subfractions, branched-chain amino acids (BCAA), and GlycA, a pro-inflammatory glycoprotein biomarker, were measured by nuclear magnetic resonance (NMR) spectroscopy. The Lipoprotein Insulin Resistance (LP-IR) score, which is based on six lipoprotein variables, was calculated.

**Results:**

In all patients, hyperaldosteronism was resolved after ADX. Body mass index and fasting plasma glucose were unchanged, but HbA1c increased (*p* = 0.002). Plasma triglycerides, large triglyceride-rich lipoprotein (TRL) cholesterol, and large TRL particles were increased (*p* < 0.01), resulting in an increase in TRL size (*p* = 0.027). High-density lipoprotein size was decreased (*p* = 0.015). LP-IR scores (*p* = 0.001) and total BCAA (*p* = 0.017) were increased, but GlycA remained unaltered.

**Conclusions:**

Based on increases in LP-IR scores and BCAA, which each have been shown to predict new onset type 2 diabetes mellitus independent of conventional risk factors in the general population, this preliminary study suggests that diabetes risk is not improved but may even be increased after ADX for APA despite remission of PA.

## Introduction

Primary aldosteronism (PA) is a well-recognized and rather frequent cause of secondary hypertension with deleterious effects on cardiometabolic health including high prevalence of Type 2 diabetes mellitus (T2D) [1–5]. Besides pro-inflammatory effects of aldosterone [6, 7], PA is likely to be associated with yet incompletely understood abnormalities in lipid and glucose metabolism [5, 8]. The majority of PA cases are due to either unilateral aldosterone-producing adenoma (APA) or bilateral adrenal hyperplasia (BAH) [9]. Laparoscopic adrenalectomy (ADX) is the preferred treatment modality for APA, and this procedure may result in relief of hyperaldosteronism in more than 90% of cases [9]. Clearly, this procedure offers the possibility to study abnormalities in lipid and glucose metabolism as part of routine follow-up of such patients.

Remarkably, three recent studies have suggested that the plasma lipoprotein profile may be worsened 1 year after unilateral ADX for PA [10–12]. In particular, an unexpected increase in plasma triglycerides (TG) was observed in all these studies [10–12]. In one of these studies, there was a small decrease in fasting plasma glucose (FPG), without a change in the classification of patients with impaired glucose tolerance or T2D mellitus [11]. Moreover, in a small group of APA patients, the first phase insulin response to intravenous glucose was diminished before treatment and increased after unilateral ADX [12]. On the other hand, we observed a small increase in glycated hemoglobin (HbA1c) after unilateral ADX [5], although insulin sensitivity, determined by the hyperinsulinemic-euglycemic clamp technique, did not significantly change after surgery [12].

During the past few years, nuclear magnetic resonance (NMR)-based techniques have been developed that allow for the high-throughput quantification of lipoprotein subfractions, glycosylated proteins, and metabolites such as branched-chain amino acids (BCAA) [13–15]. Using NMR, we observed lipoprotein abnormalities, including lower apolipoprotein B (apoB) and triglyceride-rich lipoprotein (TRL) concentrations in untreated PA patients together with higher levels of GlycA, a measure of inflammatory glycoproteins [7]. With this NMR method, the Lipoprotein Insulin Resistance (LP-IR) score has been developed, which is based on six NMR-based lipoprotein variables [15]. LP-IR scores are closely related to glucose disposal measured with the hyperinsulinemic-euglycemic clamp technique and are strongly correlated with homeostasis model assessment of insulin resistance (HOMA-IR) [15, 16]. Both the LP-IR and the plasma total BCAA concentration have been found to predict incident T2D in several population studies [16–20].

In the absence of data concerning effects of PA treatment on NMR-derived biomarkers, the present study was initiated to test the effects of unilateral ADX in APA patients on plasma lipoprotein subfractions, LP-IR, BCAA, and GlycA.

## Materials and methods

### Study population

The diagnostic work-up of the study participants was performed in accordance with the Endocrine Society Practice Guidelines [21]. Patients suspected for PA were screened using aldosterone-to-renin ratio (ARR; cut-off 12.0 ng/mU, sitting position). If elevated, antihypertensive medication was stopped for at least 1 week (e.g., angiotensin-converting enzyme inhibitors, beta blockers, calcium antagonists, and low-dose thiazides) and 4 weeks (e.g., mineralocorticoid antagonists) whenever possible, otherwise patients received drugs with minimal effect on the ARR, such as alpha-receptor (doxazosin) or calcium-channel blockers (verapamil). Thereafter all subjects were retested again. If ARR remained abnormal, confirmatory testing (e.g., sodium loading test, captopril challenge test) was performed. In case of confirmed diagnosis of PA, adrenal vein sampling was used for subtype differentiation between unilateral and bilateral disease. Abstaining from ACTH infusion, selectivity of the catheter was assumed if the cortisol concentration (using rapid intra-procedure cortisol measurement) within the adrenal vein was at least two times higher than that in the simultaneously drawn peripheral sample. The diagnosis of unilateral disease was made if the aldosterone-to-cortisol ratio (A/C-ratio) of one adrenal vein was at least four times higher than the A/C-ratio of the contralateral side [22, 23].

For the present analysis we selected 19 patients from the Munich center of the German Conn’s Registry–Else Kröner-Fresenius Hyperaldosteronism registry fulfilling the following criteria: (1) confirmed unilateral PA treated by ADX; (2) no evidence of postoperative adrenal insufficiency at 6-month follow-up according to ACTH stimulation testing; and (3) no evidence of T2D at baseline (defined as a FPG ≥ 126 mg/dl, a HbA1c value ≥6.5% (≥48 mmol/mol) or use of glucose lowering drugs) or other relevant metabolic disorders or treatment with lipid-lowering drugs. All patients were studied after an overnight fast and were re-evaluated 6 months after surgical treatment in a standardized fashion.

Blood samples were taken between 8:00 and 10:00 A.M. On each occasion, the evaluation included the collection of anthropometric data and clinical characteristics such as duration of hypertension and current medication. Blood pressure was obtained in the sitting position after at least 15 min of rest in our outpatient clinic. 24-h ambulatory blood pressure measurements were also performed before and after PA treatment. HOMA-IR was calculated as follows: (insulin fasting [μU/ml] × FPG [mg/dl])/405.

All patients gave written informed consent and the ethics committees of the University of Munich approved the protocol.

### Laboratory methods

Laboratory work-up was performed immediately after the withdrawal of blood samples, in a fasting state in sitting position. Plasma aldosterone concentration and active renin concentration were measured using the Liaison chemiluminescence assay (Diasorin) and routine parameters using standard methods in our central laboratory [11]. Creatinine, glucose, and HbA1c were measured using routine methods [11]. Estimated glomerular filtration rate (eGFR) was calculated using the Modification of Diet in Renal Disease formula. Blood samples for NMR analysis were prepared by centrifugation at 1400 × *g* for 15 min, stored at −80 °C and sent frozen to LabCorp, Morrisville, NC, USA. NMR spectra were acquired on Vantera® Clinical Analyzers from ethylene diamine tetra acetic acid plasma samples at LabCorp as described in [16]. The *NMR MetaboProfile* analysis, which reports concentrations of lipids, apolipoproteins, lipoprotein particles and sizes, and BCAA, was performed using a recently developed deconvolution algorithm [15, 16]. Linear regression of the lipoprotein subclass signal areas against serum lipid and chemically measured apolipoprotein levels in a large study population provided the conversion factors to generate concentrations of total cholesterol (TC), TG, low-density lipoprotein cholesterol (LDL-C) and high-density lipoprotein cholesterol (HDL-C), apoB and apolipoprotein A-I. The inter-assay precision for these parameters ranges from 1.4 to 6.2%. The diameters for the lipoprotein classes reported by the LP4 algorithm are TRL particles (TRL-P) (24–240 nm) (subdivided into very large, large, medium, small and very small TRL-P), LDL particles (LDL-P) (19–23 nm) (subdivided into large, medium and small LDL-P), and HDL particles (HDL-P) (7.4–12.0 nm) (subdivided into large, medium and small HDL-P). Inter-assay precision for TRL-P, LDL-P, and HDL-P are 6.4%, 1.5%, and 2.4%, respectively. Mean TRL, LDL, and HDL particle sizes are weighted averages derived from the sum of the diameter of each subclass multiplied by its relative mass percentage. The LP-IR score was calculated using six NMR-measured lipoprotein variables: weighted average sizes of TRL, LDL, and HDL, combined with concentrations of large TRL-P, small LDL-P and large HDL-P [15]. LP-IR scores vary between 0 and 100; the higher the score the more insulin resistant the individual [15, 16]. Details for the quantification of the BCAA have been reported previously [19]. The inter-assay precisions are 3.1% for valine, 5.9% for leucine 14.1% for isoleucine, and 3.2% for total BCAA. The GlycA signal was quantified as described in [24, 25]. The GlycA NMR signal originates from highly mobile protons of *N*-acetylglucosamine residues located on the carbohydrate side-chains of circulating acute phase proteins (e.g., α1-acid glycoprotein, haptoglobin, α1-antitrypsin, α1-antichymotrypsin, and transferrin). The signals from these *N*-acetylglucosamine residues were used to calculate the concentrations of GlycA (µmol/L). The intra-assay and inter-assay precisions for GlycA are 1.9% and 2.6%, respectively.

### Statistical analysis

Data analysis was performed using IBM SPSS software (version 25.0, SPSS Inc., USA). Results are given in median and 25th and 75th percentile if not mentioned otherwise. body mass index (BMI) was calculated as weight in kilograms divided by the square of the height in meters. Comparisons in variables before and after ADX were performed using Wilcoxon singed-rank test tests for paired observations. Spearman’s Rank Order test was used to determine the relationships between variables. Two-sided *p* values < 0.05 were considered to be statistically significant.

## Results

Ten men and nine women participated (median age 46 years). All had at baseline high aldosterone levels and elevated blood pressure, as expected. Adrenal-venous sampling pointed to unilateral disease in all of them, with a right sided APA in ten and a left sided APA in nine patients. Imaging techniques confirmed the lateralization results in 10 of 19 cases. There was only one patient with contralateral mass as well as eight patients without any adrenal mass detected by imaging. Adrenal mass size was in median 10 mm and ranged from 2 to 22 mm.

Parameters of renal function as well as FPG and other parameters of glucose metabolism were within normal ranges (Table 1). With a median BMI of 25.6 kg/m^2^ patients were slightly overweight (Table 1). Six patients had a history of nicotine use whereas three of them were current smokers. Twelve patients consumed moderate amounts of alcohol while seven patients were abstainers.

**Table 1:** Clinical characteristics, plasma aldosterone, renin, potassium, and renal function in 19 patients with primary aldosteronism due to unilateral aldosterone-producing adenoma

Patient characteristics (*n* = 19)	*n*	Before ADX	After ADX	*p* value
Gender [f/m]	19	9/10	–	
Age [years]	19	46 [41; 56]	–	
Adrenal mass size [mm]	11	10 [8; 19]	–	
BMI [kg/m^2^]	19	25.6 [23.8; 29.4]	26.2 [23.8; 30.7]	0.583
Aldosterone [ng/l]	19	189 [129; 270]	71[49; 115]	**0.000**
Plasma renin [mU/l]	19	4.0 [2.0; 6.5]	8.6 [6.2; 14,8]	**0.001**
SBP [mmHg]	19	153 [131; 167]	140 [129; 155]	0.126
DBP [mmHg]	19	91 [83; 97]	96 [87; 102]	0.268
24-h SBP [mmHg]	12	153 [148; 158]	127 [119; 132]	**0.018**
24-h DBP [mmHg]	12	98 [93; 105]	79 [74; 86]	**0.018**
DDD of antihypertensive medication [n]	19	2.0 [1.3; 3.0]	0.0 [0.0; 0.5]	**0.002**
Serum potassium [mmol/l]	19	3.5 [3.3; 3.9]	4.2 [4.0; 4.3]	**0.000**
Serum creatinine [mg/dl]	19	0.9 [0.8; 1.0]	0.9 [0.8; 1.0]	**0.029**
eGFR [ml/min/1.73 m^2^]	19	81 [75; 93]	77 [65; 94]	**0.013**
FPG [mg/dl]	19	97 [91; 102]	96 [89; 103]	0.678
Insulin [μU/ml]	19	5.6 [4.2; 11.5]	n.a.	
HOMA-IR [µU/ml × FPG [mg/dl])/405]	19	1.2 [0.9; 2.4]	n.a.	
HbA1c [%]	19	5.1 [5.0; 5.5]	5.4 [5.2; 5.7]	**0.002**

Data before and 6 months after adrenalectomy are shown

Continuous data are given as median (interquartile) range

Comparisons were performed by Wilcoxon signed rank tests. Statistically significant changes are marked in bold. To convert creatinine from mg/dl to µmol/l multiply by 88.4. To convert glucose from mg/dl to mmol/l multiply by 0.056. To convert HbA1c from % to mmol/mol multiply by 8.5

*ADX* adrenalectomy, *DDD* defined daily dose, *DBP* diastolic blood pressure, *eGFR* estimated glomerular filtration rate, *FPG* fasting plasma glucose, *n.a.* not available, *SBP* systolic blood pressure

Six months after unilateral ADX, aldosterone and renin levels were normalized (Table 1). Outpatient clinic systolic and diastolic blood pressure did not change significantly, but defined daily doses of antihypertensives as well as ambulatory 24-h systolic and diastolic blood pressure had reduced significantly. Expectedly, eGFR was reduced, consequent to reduced renal plasma flow after PA treatment (Table 1). BMI and FPG remained unaltered but HbA1c increased slightly after ADX. Postoperative plasma insulin levels were not available. Plasma TC and LDL-C were unchanged, whereas apoB levels tended to increase. Plasma TG increased, coinciding with an increase in TRL cholesterol. This increase in TG was attributable to an increase in large TRL-P and resulted in an increase in TRL size (Table 2). Although HDL-C and HDL-P did not change, small HDL-P increased, resulting in a decrease in HDL size (Table 2). Consequent to these changes in TRL and HDL subfractions, LP-IR scores increased (Table 2). This increase in LP-IR tended to be correlated with the increase in HbA1c (*r* = 0.352, *p* = 0.14) and was significantly correlated with the decrease in eGFR (*r* = −0.466, *p* = 0.044). In addition, we observed a positive correlation of LP-IR scores with HOMA-IR at baseline (*r* = 0.543, *p* = 0.016).

**Table 2:** Plasma glucose, lipids, apolipoproteins, lipoprotein subfractions, lipoprotein sizes, the Lipoprotein insulin resistance (LP-IR) score, branched-chain amino acids (BCAA), and GlycA in 19 patients with primary aldosteronism due to unilateral aldosterone-producing adenoma

Patient characteristics (*n* = 19)	Before ADX	After ADX	*p* value
Total cholesterol [mg/dl]	158 [146; 169]	169 [151; 187]	0.142
HDL-C [mg/dl]	49 [42; 66]	45 [41; 61]	0.355
LDL-C [mg/dl]	87 [67; 105]	99 [69; 112]	0.456
Triglycerides [mg/dl]	84 [73; 106]	108 [87; 133]	**0.004**
TRL-TG [mg/dl]	45 [42; 63]	66 [57; 90]	**0.009**
TRL-C [mg/dl]	19 [16; 23]	25 [18; 33]	**0.040**
ApoB [mg/dl]	76 [59; 85]	89 [59; 98]	0.098
ApoA-I [mg/dl]	129 [116; 146]	127 [119; 147]	0.896
TRL-P [nmol/l]	122 [84; 149]	124 [105; 192]	0.136
Very large TRL-P [nmol/l]	0.0 [0.0; 0.1]	0.0 [0.0; 0.1]	0.227
Large TRL-P [nmol/l]	1.0 [0.0; 3.6]	2.8 [1.6; 5.4]	**0.007**
Medium TRL-P [nmol/l]	6.0 [3.7; 9.5]	9.9 [3.7; 17.0]	0.077
Small TRL-P [nmol/l]	34 [21; 48]	29.8 [8.5; 78.3]	0.904
Very small TRL-P [nmol/l]	78 [29; 100]	77.9 [35.5; 126.8]	0.546
LDL- P [nmol/l]	1246 [955; 1490]	1473 [991; 1625]	0.153
Large LDL-P [nmol/l]	510 [419; 706]	457 [364; 570]	0.094
Medium-LDL-P [nmol/l]	269 [88; 560]	232 [0; 466]	0.546
Small LDL-P [nmol/l]	412 [194; 511]	389 [222; 807]	0.212
HDL-P [µmol/l]	20 [18; 22]	21 [18; 23]	0.231
Large HDL-P [µmol/l]	2.2 [0.9; 3.1]	1.5 [0.9; 3.4]	0.323
Medium HDL-P [µmol/l]	5.1 [3.7; 6.6]	4.8 [3.9; 6.0]	0.235
Small HDL-P [µmol/l]	12.4 [11.3; 14.3]	14.4 [12.3; 15.7]	**0.038**
TRL size [nm]	43.4 [39.6; 53.4]	48.1 [42.2; 61.4]	**0.027**
LDL size [nm]	21.4 [21.3; 21.7]	21.4 [21.1; 21.7]	0.346
HDL size [nm]	9.1 [8.8; 9.7]	8.9 [8.5; 9.3]	**0.015**
LP-IR score	30 [15; 51]	54 [20; 63]	**0.001**
Total BCAA [µmol/l]	334 [277; 374]	386 [299; 447]	**0.017**
Valine [µmol/l]	187 [177; 195]	209 [171; 232]	**0.016**
Leucine [µmol/l]	100 [76; 114]	116 [79; 135]	0.117
Isoleucine [µmol/l]	49 [35; 60]	55 [40; 68]	**0.011**
GlycA [µmol/l]	329 [279; 367]	333 [301; 378]	0.587

Data before and 6 months after adrenalectomy are shown

Continuous data are given as median (interquartile) range

Comparisons were performed by Wilcoxon signed rank tests. Statistically significant changes are marked in bold. To convert cholesterol form mg/dl to mmol/l multiply by 0.02586. To convert triglycerides from mg/dl to mmol/l multiply by 0.01129

*ADX* adrenalectomy, *Apo* apolipoprotein, *BCAA* branched-chain amino acids, *HDL* high-density lipoproteins, *HDL-P* HDL particles, *LDL* low-density lipoproteins, *LDL-C* LDL cholesterol, *LDL-P* LDL particles, *TRL* triglyceride-rich lipoproteins, *TRL-P* TRL particles

Of further note, plasma total BCAA increased after ADX, mainly due to increases in valine and isoleucine. This increase in total BCAA was not significantly related to the decrease in eGFR (*r* = 0.144, *p* = 0.556) or the increase in LP-IR scores (*r* = 0.322, *p* = 0.178). Plasma GlycA did not change after ADX (Table 2).

## Discussion

In this preliminary study, we present for the first time results of NMR-measured lipoprotein subfractions and other targeted metabolomics biomarkers before and after unilateral ADX for APA. First, the current study shows that plasma TG are increased after ADX consequent to a rise in large TRLs. As a result, TRL size increased, and these changes were accompanied by a shift in HDL particle distribution with an increase in small HDL-P and a decrease in HDL particle size. Consequently, the LP-IR score, an NMR-based measure of insulin resistance, increased. Of further note, we observed a hitherto unreported rise in total plasma BCAA after ADX. Given that the LP-IR scores [16–18] and BCAA concentrations [20] have been shown to predict incident diabetes in population-based cohort studies, our present findings suggest that the risk of future diabetes development is not ameliorated and could even be worsened in PA patients without T2D initially despite remission of hyperaldosteronism after unilateral ADX.

The current results regarding the increase in plasma TG after ADX agree with and expand on previous publications showing an increase in TG after PA treatment [10–12]. In comparison, HDL-C tended to decrease, irrespective of the presence of dyslipidemia before surgery in a Japanese study [10]. We documented a drop in HDL-C 1 year after unilateral ADX in our earlier German study [11]. In the latter report, similar lipoprotein changes were observed in patients with BAH after mineralocorticoid receptor antagonist (MRA) treatment [11]. A recent study from the German Conn registry also reported a decrease in HDL-C after unilateral ADX [5]. In the present study, HDL-C and HDL-P remained unchanged but HDL size was decreased coinciding with an increase in large TRLs, which is conceivably attributable at least in part to effects of TRLs on HDL metabolism via the cholesteryl ester transfer process [26]. We did not observe significant increases in LDL-C and LDL-P after ADX, but the trends observed were comparable with those in the Japanese study which showed an increase in LDL-C after PA treatment [10]. The mechanisms responsible for plasma TRL increments after ADX for PA are not completely understood. In the previous reports, the development of dyslipidemia and changes in TG and HDL-C were found to be dependent on (changes) eGFR after treatment [10, 11]. In line, we observed that changes in LP-IR scores were correlated with changes in eGFR. However, the decline in eGFR after ADX as observed in the current study was modest, amounting to about 5%, which makes a contribution of a decline in kidney function on plasma lipoprotein alterations uncertain. Moreover, HDL-C is inversely rather than directly correlated with eGFR in subjects without chronic kidney disease [27], questioning whether such a limited decline in kidney function may explain a decrease in HDL-C after ADX. Of further relevance, co-secretion of cortisol in PA and relative adrenal insufficiency may affect glucose and lipid metabolism after ADX [5]. Notably, in all the cases included in the present series, adrenal function had recovered 6 months after ADX. Finally, it should be appreciated that the present study was carried out along with routine clinical treatment for PA due to APA. As a consequence, antihypertensive treatment was extensively reduced after surgery. Hence, we cannot exclude that cessation of certain drugs like MRA or doxazosin could have influenced lipid metabolism [28–30]. Yet, we found that MRA in case of bilateral disease elicits comparable changes in TG and HDL-C compared with ADX for APA [11].

The LP-IR signature reflects the multifaceted aspects of insulin resistance on lipoprotein metabolism [18]. The LP-IR index is robustly correlated with HOMA-IR [16], a finding which was reinforced in APA patients. Despite its strong relationship with HOMA-IR [16, 17], LP-IR scores predicted incident T2D even when taking account of HOMA-IR [16, 17]. Notably, LP-IR improves diabetes risk classification beyond the Framingham Offspring risk score [16, 17], and it has been proposed that LP-IR may reflect early insulin resistant dyslipidemia before development of hyperglycemia [18]. Hence, it could be proposed that the higher LP-IR scores after ADX represents an early marker of elevated diabetes risk before a rise in FPG becomes evident. Accumulating evidence supports the idea that BCAA are important in the pathogenesis of dysglycemia [31]. BCAA may disrupt the function of the mammalian target of rapamycin complex 1, which subsequently leads to insulin resistance and oxidative stress [32, 33]. In view of the recent findings demonstrating that plasma BCAA predicts new onset T2D [19] we surmise that higher total BCAA could translate into increased diabetes risk after ADX. Taken together, we postulate that two, at least in part, independent pathways are operating that could pose patients with biochemical remission of PA at increased risk for diabetes development after ADX.

Finally, we did not observe that GlycA decreased after ADX. This result suggests that low-grade systemic inflammation is unaffected by ADX, and is unexpected in view of the recent results showing higher GlycA levels in untreated PA patients compared with healthy subjects and subjects with (un)treated hypertension [7].

The current study was conducted in a limited number of patients, we consider the present findings preliminary. Moreover, we did not follow adrenal incidentaloma patients without evidence of hormonal hypersecretion as a control group to determine variations in NMR-derived biomarkers over time. However, our prospective observations in a rather homogeneous group of PA patients whose hyperaldosteronism was remitted biochemically after ADX can be seen as a strength of the present study. Nonetheless, it is evident that longitudinal studies with prolonged follow-up in larger patient groups are required to determine whether the long-term risk of new onset T2D is indeed elevated in this patient category.

In conclusion, based on an increase in both the LP-IR index and BCAA, our current findings suggest that diabetes risk is not improved but may even be increased after ADX for APA despite remission of PA in patients without T2D initially.

## References

[1] Reincke M, Meisinger C, Holle R, Quinkler M, Hahner S, Beuschlein F, Bidlingmaier M, Seissler J, Endres S (2010). Participants of the German Conn’s Registry. Is primary aldosteronism associated with diabetes mellitus? Results of the German Conn’s Registry. Horm. Metab. Res..

[2] Remde H, Hanslik G, Rayes N, Quinkler M (2015). Glucose metabolism in Primary Aldosteronism. Horm. Metab. Res..

[3] Reincke M, Beuschlein F (2015). Progress in primary aldosteronism: translation on the move. Horm. Metab. Res..

[4] Monticone S, D’Ascenzo F, Moretti C, Williams T, Veglio F, Gaita F, Mulatero P (2018). Cardiovascular events and target organ damage in primary aldosteronism compared with essential hypertension: a systematic review and meta-analysis. Lancet Diabetes Endocrinol..

[5] Gerards J, Heinrich D, Adolf C, Meisinger C, Rathmann W, Sturm L, Nirschl N, Bidlingmaier M, Beuschlein F, Thorand B (2019). Impaired glucose metabolism in primary aldosteronism is associated with cortisol cosecretion. J. Clin. Endocrinol. Metab..

[6] Farquharson C, Struthers A (2002). Aldosterone induces acute endothelial dysfunction in vivo in humans: evidence for an aldosterone-induced vasculopathy. Clin. Sci..

[7] Berends A, Buitenwerf E, Gruppen E, Sluiter W, Bakker S, Connelly M, Kerstens M, Dullaart R (2019). Primary aldosteronism is associated with decreased low-density and high-density lipoprotein particle concentrations and increased GlycA, a pro-inflammatory glycoprotein biomarker. Clin. Endocrinol..

[8] Krug A, Ehrhart-Bornstein M (2008). Aldosterone and metabolic syndrome: is increased aldosterone in metabolic syndrome patients an additional risk factor?. Hypertension.

[9] Williams T, Lenders J, Mulatero P, Burrello J, Rottenkolber M, Adolf C, Satoh F, Amar L, Quinkler M, Deinum J (2017). Primary Aldosteronism Surgery Outcome (PASO) investigators. Outcomes after adrenalectomy for unilateral primary aldosteronism: an international consensus on outcome measures and analysis of remission rates in an international cohort. Lancet Diabetes Endocrinol..

[10] Kaga M, Utsumi T, Tanaka T, Kono T, Nagano H, Kawamura K, Kamiya N, Imamoto T, Nihei N, Naya Y (2015). Risk of new-onset dyslipidemia after laparoscopic adrenalectomy in patients with primary aldosteronism. World J. Surg..

[11] Adolf C, Asbach E, Dietz A, Lang K, Hahner S, Quinkler M, Rump L, Bidlingmaier M, Treitl M, Ladurner R (2016). Worsening of lipid metabolism after successful treatment of primary aldosteronism. Endocrine.

[12] Fischer E, Adolf C, Pallauf A, Then C, Bidlingmaier M, Beuschlein F, Seissler J, Reincke M (2013). Aldosterone excess impairs first phase insulin secretion in primary aldosteronism. J. Clin. Endocrinol. Metab..

[13] Connelly M, Gruppen E, Otvos J, Dullaart R (2016). Inflammatory glycoproteins in cardiometabolic disorders, autoimmune diseases and cancer. Clin. Chim. Acta.

[14] Würtz P, Kangas A, Soininen P, Lawlor D, Davey Smith G, Ala-Korpela M (2017). Quantitative serum nuclear magnetic resonance metabolomics in large-scale epidemiology: a primer on -omic technologies. Am. J. Epidemiol..

[15] Shalaurova I, Connelly M, Garvey W, Otvos J (2014). Lipoprotein insulin resistance index: a lipoprotein particle-derived measure of insulin resistance. Metab. Syndr. Relat. Disord..

[16] Flores-Guerrero J, Connelly M, Shalaurova I, Gruppen E, Kieneker L, Dullaart R, Bakker S (2019). Lipoprotein insulin resistance index, a high-throughput measure of insulin resistance, is associated with incident type II diabetes mellitus in the prevention of renal and vascular end-stage disease study. J. Clin. Lipido..

[17] Mackey R, Mora S, Bertoni A, Wassel C, Carnethon M, Sibley C, Goff D (2015). Lipoprotein particles and incident type 2 diabetes in the multi-ethnic study of atherosclerosis. Diabetes Care..

[18] Harada P, Demler O, Dugani S, Akinkuolie A, Moorthy M, Ridker P, Cook N, Pradhan A, Mora S (2017). Lipoprotein insulin resistance score and risk of incident diabetes during extended follow-up of 20 years: the Women’s Health Study. J. Clin. Lipido..

[19] Wolak-Dinsmore J, Gruppen E, Shalaurova I, Matyus S, Grant R, Gegen R, Bakker S, Otvos J, Connelly M, Dullaart R (2018). A novel NMR-based assay to measure circulating concentrations of branched-chain amino acids: Elevation in subjects with type 2 diabetes mellitus and association with carotid intima media thickness. Clin. Biochem..

[20] J. Flores-Guerrero, M. Osté, L. Kieneker, E. Gruppen, J. Wolak-Dinsmore, J. Otvos, M. Connelly, S. Bakker, R. Dullaart, Plasma branched-chain amino acids and risk of incident type 2 diabetes: results from the PREVEND Prospective Cohort Study. J. Clin. Med. **7** (2018). 10.3390/jcm7120513 10.3390/jcm7120513 PMC6306832 30518023

[21] Funder J, Carey R, Mantero F, Murad M, Reincke M, Shibata H, Stowasser M, Young W (2016). The management of primary aldosteronism: case detection, diagnosis, and treatment: an endocrine society clinical practice guideline. J. Clin. Endocrinol. Metab..

[22] Betz M, Degenhart C, Fischer E, Pallauf A, Brand V, Linsenmaier U, Beuschlein F, Bidlingmaier M, Reincke M (2011). Adrenal vein sampling using rapid cortisol assays n primary aldosteronism is useful in centers with low success rates. Eur. J. Endocrinol..

[23] Ladurner R, Sommerey S, Buechner S, Dietz A, Degenhart C, Hallfeldt K, Gallwas J (2017). Accuracy of adrenal imaging and adrenal venous sampling in diagnosing unilateral primary aldosteronism. Eur. J. Clin. Investig..

[24] Otvos J, Shalaurova I, Wolak-Dinsmore J, Connelly M, Mackey R, Stein J, Tracy R (2015). GlycA: a composite nuclear magnetic resonance biomarker of systemic inflammation. Clin. Chem..

[25] E. Gruppen, S. Kunutsor, L. Kieneker, B. van der Vegt, M. Connelly, G. de Bock, R. Gansevoort, S. Bakker, R. Dullaart, GlycA, a novel pro-inflammatory glycoprotein biomarker is associated with mortality: results from The PREVEND study and meta-analysis. J. Intern. Med. (2019). 10.1111/joim.12953 10.1111/joim.12953 PMC6851697 31260573

[26] Dullaart R, Dallinga-Thie G, Wolffenbuttel B, van Tol A (2007). CETP inhibition in cardiovascular risk management: a critical appraisal. Eur. J. Clin. Investig..

[27] Krikken J, Gansevoort R, Dullaart R, PREVEND Study Group (2010). Lower HDL-C and apolipoprotein A-I are related to higher glomerular filtration rate in subjects without kidney disease. J. Lipid Res..

[28] Hirano T, Yoshino G, Kashiwazaki K, Adachi M (2001). Doxazosin reduces prevalence of small dense low density lipoprotein and remnant-like particle cholesterol levels in nondiabetic and diabetic hypertensive patients. Am. J. Hypertens..

[29] Iwamoto N, Abe-Dohmae S, Ayaori M, Tanaka N, Kusuhara M, Ohsuzu F, Yokoyama S (2007). ATP-binding cassette transporter A1 gene transcription is downregulated by activator protein 2alpha. Doxazosin inhibits activator protein 2alpha and increases high-density lipoprotein biogenesis independent of alpha1-adrenoceptor blockade. Circ. Res..

[30] Srivastava A, Adams-Huet B, Vega G, Toto R (2016). Effect of losartan and spironolactone on triglyceride-rich lipoproteins in diabetic nephropathy. J. Investig. Med..

[31] Lynch C, Adams S (2014). Branched-chain amino acids in metabolic signaling and insulin resistance. Nat. Rev. Endocrinol..

[32] Batch B, Shah S, Newgard C, Turer C, Haynes C, Bain J, Muehlbauer M, Patel M, Stevens R, Appel L (2013). Branched chain amino acids are novel biomarkers for discrimination of metabolic wellness. Metabolism.

[33] M. Yoon, The emerging role of branched-chain amino acids in insulin resistance and metabolism. Nutrients. (2016). 10.3390/nu8070405 10.3390/nu8070405 PMC4963881 27376324

